# Factors that interfere the medication compliance in hypertensive patients

**DOI:** 10.1590/S1679-45082013000300012

**Published:** 2013

**Authors:** Ana Carolina Queiroz Godoy Daniel, Eugenia Velludo Veiga

**Affiliations:** 1 Hospital Israelita Albert Einstein, São Paulo, SP, Brazil; 2 Universidade de São Paulo, Ribeirão Preto, SP, Brazil

**Keywords:** Patient compliance, Hypertension/therapy, Hypertension/pharmacology, Treatment refusal

## Abstract

**Objective:**

To characterize the factors that interfere in drug treatment compliance in a group of individuals with arterial hypertension.

**Methods:**

A non-experimental descriptive study that analyzed a sample of 80 patients diagnosed with arterial hypertension, who underwent medical treatment and were admitted to a university hospital during the period from March to May 2009. To collect data, the Instrument for Evaluation of Attitudes Regarding Taking Medication was applied.

**Results:**

In the studied population, 45.1% had sufficient degree of compliance to drug therapy. Individuals with controlled blood pressure, females, white, single, married or widowed, retired, aged between 40 and 59 years, and those aged above 80 years were the interviewees who answered positively regarding compliance and follow-up of drug therapy.

**Conclusion:**

Despite the fact that the number of factors that facilitate the process of compliance to drug treatment is greater than the number of complicating factors, we found that more than half of the patients surveyed had an insufficient degree of compliance with drug treatment for high blood pressure, which demonstrates the need to develop studies aimed to identify these factors and their contribution to the promotion of patient autonomy, acceptance, awareness and adaptation regarding their illness.

## RESUMO

**Objetivo:**

Caracterizar os fatores que interferem na adesão terapêutica medicamentosa de portadores de hipertensão arterial.

**Métodos:**

Estudo de caráter descritivo, de natureza não experimental, que analisou uma amostra de 80 pacientes com diagnóstico de hipertensão arterial submetidos a tratamento medicamentoso e internados em um hospital universitário, no período de março a maio de 2009. Para a obtenção dos dados, foi aplicado o Instrumento para Avaliar Atitudes Frente à Tomada dos Remédios.

**Resultados:**

Dentre a população estudada, 45,1% apresentaram suficiente grau de adesão à terapêutica medicamentosa. Indivíduos com pressão arterial controlada, gênero feminino, brancos, solteiros, casados ou viúvos, aposentados, de faixa etária entre 40 e 59 anos e aqueles com idade igual ou superior a 80 anos foram os entrevistados que responderam de maneira positiva ao cumprimento e seguimento da terapêutica medicamentosa.

**Conclusão:**

Apesar do número de fatores facilitadores ao processo de adesão à terapêutica medicamentosa ser maior do que o número de fatores dificultadores, observou-se que mais da metade dos pacientes entrevistados apresentou insuficiente grau de adesão à tomada de medicamentos para a hipertensão arterial. Isso demonstra a necessidade de desenvolvimento de estudos voltados à identificação desses fatores, bem como sua contribuição para a promoção de autonomia, aceitação, conhecimento e adaptação do paciente em relação à sua própria doença.

## INTRODUCTION

Arterial hypertension (AH) is characterized by sustained increases in systemic blood pressure. Chronically, this elevation is associated with a higher risk of renal, cardiac, and brain damage, as well as with other diseases^(1)^. The control of AH aims to reduce cardiovascular morbidity and mortality. Drug treatment and non-drug treatment contribute to maintaining arterial blood pressure (BP) below 140mmHg for systolic pressure (SBP) and 90mmHg for diastolic pressure (DBP)^(2)^.

Although making the diagnosis may considered easy and efficient treatment measures exist, the effective maintenance and control of the therapeutic regimen related to AH has been an arduous task. This situation has been experienced by the individuals affected by the condition, their family members, healthcare professionals and organizations.

The expression “compliance to treatment” refers to the degree of obedience to the indicated treatment measures, whether drug-based or not, with the objective of maintaining the BP at normal levels. Compliance is a complex behavioral process, strongly influenced by the environment, by healthcare professionals and by medical care^(3)^. Compliance is positive, necessary, and should occur in 100% of hypertensive individuals submitted to treatment.

In clinical practice, a significant discontinuation of drug treatment is noted, and accounts for 16 to 50% of abandonment during the first year of treatment^(4)^.

Drug therapy of the individual with AH is, in most cases, indispensable for reducing and maintaining control of pressure levels. Additionally, many times it is accompanied by undesirable effects of treatment, having been prescribed for a long term and having an elevated cost. The medications prescribed should enable the process, but become complicating factors for treatment, which often compromises compliance itself and does not guarantee the reduction of BP values, interfering in control of the disease, prevention of complications, and in worsening of the disease.

To prioritize awareness about the factors that interfere in compliance may contribute towards the development of innovative strategies that might encourage patients to be motivated by the treatment; as well as contribute with healthcare professionals in planning, execution, and evaluation of care given; and guarantee an effective health policy in the control of AH at the different levels of complexity.

## OBJECTIVE

To characterize the factors that interfere in drug treatment compliance in a group of individuals with arterial hypertension.

## METHODS

This is a descriptive non-experimental study that combined information on phenomena that characterize factors with positive (facilitators) and negative (complicators) interference of the process of drug treatment compliance in patients with AH. From a population of 369 patients, 69 were selected, aged between 40 and 80 years, of both genders, who agreed to voluntarily participate in the study and signed the informed consent form. All of them had diagnosis of AH, were submitted to drug treatment, and were hospitalized at the Clinical Unit, *Hospital das Clínicas da Faculdade de Medicina de Ribeirão Preto da Universidade de São Paulo,* during the period of March to May 2009. The project was submitted to the Research Ethics Committee of this institution and was approved, under official statement number 3.834/2008.

Patients with a diagnosis of AH in serious condition, with physical and psychic difficulties to participate in the interview, or who refused to participate voluntarily in the study were excluded from the study.

To gather data relative to factors that interfere in compliance with drug treatment in individuals with AH, an instrument for data collection with structured questions was applied to the patients, which encouraged grouping of personal information. The data were obtained by means of the interview and the form for health evaluation.

The data collection instrument was prepared in two parts: the first consisted of the identification, biology, and environment of the individual, with open and closed questions. The second portion covered the Instrument to Evaluate Attitudes Regarding Taking Medication (IAAFTR); this was prepared based on the professional experience of Strelec^(5)^, along with patients with AH treated at a Primary Care Unit (UBS, acronym in Portuguese) in the municipality of Mogi das Cruzes (SP). It consisted of ten closed and structured questions, with affirmative or negative responses related to the theme of the study (Chart 1).

**Chart 1:** Instrument to Evaluate Attitudes Regarding Taking Medication

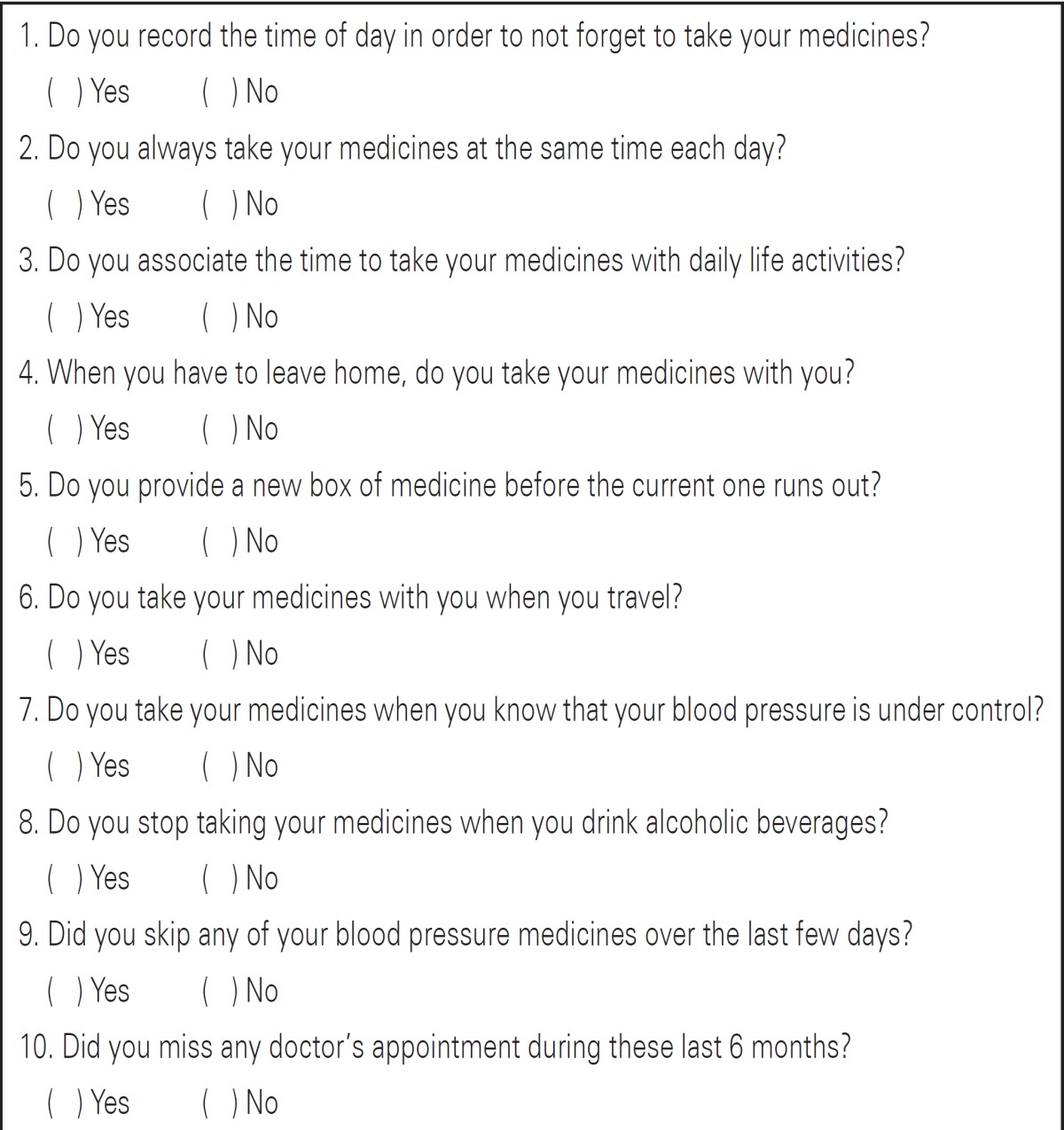

Persons considered hypertensive were those with at least one of the following criteria: diagnosis of AH described in medical records, use of antihypertensive treatment, and blood pressure levels ≥140/90mmHg. Blood pressure measurement was made as per recommendations of the 6^th^ Brazilian Guidelines for Arterial Hypertension ^(2)^.

After collection, data were organized in a computerized database and then processed and analyzed in a qualitative and quantitative form.

## RESULTS

The patients studied were characterized by a predominance of females, white, older than 50 years of age, married, with a low level of schooling and low income, and retired. As to body mass index (BMI), 65.2% of the individuals were overweight. In the same way, when measuring the abdominal circumference of the hospitalized patients, 60.9% of the men had a waist measurement >102cm and 84.8% of the women measured >88cm, values considered borderline by the 1^st^ Brazilian Guidelines for Diagnosis and Treatment of Metabolic Syndrome^(6)^.

Of the patients assessed, more than 70% had controlled SBP and DBP (up to 139/89mmHg) at the time of the interview, considering hospitalization as a facilitating element for the control of AH.

A large part of those interviewed (84.1%) had AH for 5 years or more, characterizing the chronicity of the disease as a profile of the group of hypertensive individuals studied (Table 1).

**Table 1:** Biosocial characteristics of hypertensive individuals

Variables		n (%)
Gender		
	Female	46 (66.7)
	Male	23 (33.3)
Race		
	White	55 (79.7)
	Non-white	14 (20.3)
Marital status		
	Married	42 (60.9)
	Widowed	12 (17.4)
	Separated	11 (15.9)
	Single	4 (5.8)
Schooling		
	Illiterate	15 (21.7)
	Elementary School	44 (63.8)
	Secondary School	6 (8.7)
	Higher Education	4 (5.8)
Occupation		
	Housekeeping	22 (31.9)
	Retired	32 (46.4)
	Others	15 (21.7)
Family income (minimum wages)		
	<1	22 (31.9)
	1 a 2	26 (37.7)
	2 a 3	9 (13.0)
	>3	12 (17.4)
Age (years, mean±sd)		64.01 ± 9.9
Waist (cm, mean±sd)		104.42±20.9
Body mass index (kg/m2, mean±sd)		39.398±11.2
Arterial blood pressure		
Systolic pressure (mmHg, mean±sd)		125.75±23.5
Diastolic pressure (mmHg, mean±sd)		74.66±13.5

As to evaluation of attitudes regarding taking medications, it was noted that more than half of the patients responded positively to the questionnaires on taking medication for AH always at the same time; taking medications correctly when having to leave the house; providing a new box of medicine before the previous box ran out; taking the medications along on every trip; taking the medications when the BP was controlled; not skipping the medications when there was ingestion of alcohol; and not quitting one of the medications for AH in the last few days.

As to the questionnaires about missing the medical visit over the last 6 months; recording the time of day when the AH medications were taken, and associating taking the drug with regular day-to-day activities; more than half of the patients submitted to the data collection instrument responded negatively to drug treatment compliance (Table 2).

**Table 2:** Distribution of patients with arterial hypertension as per responses collected to evaluate the attitude as to taking the medications

Questions	Responses	n (%)
Do you make a note of the time of day in order to not forget to take your medicines?	Yes	12 (17.4)
	No	57 (82.6)
2. Do you take your medicines always as the same time?	Yes	36 (52.2)
	No	33 (47.8)
3. Do you associate the time to take your medicines with your daily activities?	Yes	21 (30.4)
	No	48 (69.6)
4. When you have to leave your house, do you take your medicines along with you?	Yes	60 (86.9)
	No	9 (13.1)
5. Do you provide a new box of medicines before the previous one runs out?	Yes	54 (78.3)
	No	15 (21.7)
6. Do you take your medicines with you when you travel?	Yes	67 (97.1)
	No	2 (2.9)
7. Do you take your medicines when you know that your blood pressure is under control?	Yes	69 (100)
	No	0
8. Do you stop taking your medicines when you drink alcoholic beverages?	Yes	5 (7.2)
	No	64 (92.8)
9. Did you quit taking any of the medicines for blood pressures over the last few days?	Yes	8 (11.6)
	No	61 (88.4)
10. Did you miss any visits to your doctor over these last 6 months ?	Yes	19 (27.5)
	No	50 (72.5)

Source: Pierin AM, Strelec MA, Mion DJ. The influence of patient's consciousness regarding high blood pressure and patient's attitude in face of disease-controlling medicine intake. Arq Bras Cardiol. 2003;81(4):343-54.

In figuring the sum of points obtained by IAAFTR, it was noted that 91.30% of the patients reached scores between 6 and 9 points, and that only 1.45% had a maximal score of 10 points for the questions. No patient had scores between 0 and 1, which represents, in the survey, minimal treatment compliance with antihypertensive agents. Compliance with drug treatment was considered sufficient in those patients that reached scores of 8 or more points, which represented 44.93% of all those interviewed.

Interfering factors in the process of drug treatment compliance were those that can act positively (facilitating factors) or negatively (complicating factors) on the correct treatment follow-up.

Didactically, facilitating factors are those that contribute towards better patient compliance with their drug treatment, allowing treatment follow-up and management as per guidelines established by the healthcare team. On the other hand, complicating factors were understood as those that impede correct treatment follow-up or that hinder the incorporation of positive behaviors of the patient regarding correct drug treatment compliance^(7)^.

In this way, the main factors that interfere in drug treatment compliance are enumerated, considering the characterization data of the sample studied and the responses obtained in the proposed questionnaire (Chart 2).

**Chart 2:** Distribution of facilitating and complicating factors in the drug treatment compliance process in patients with arterial hypertension

Facilitating factors		Complicating factors	
1	Association between taking medication and daily activities	1	Forgetting to take medications
2	Recording of the times of day to take the medications	2	Side effects of the medications
3	Providing a new box of medication before the previous one is empty	3	Complexity of the therapeutic regimen
4	Habit of the patient carrying the medications with him/her	4	High cost of the medications
5	Always taking the medications at the same time of day	5	Lack of access to antihypertensive medication
6	Understanding of the disease and treatment	6	Time of treatment
7	Persistence in treatment	7	Fear of using medications along with alcoholic beverages
8	Acceptance of treatment	8	Insecurity as to the treatment
9	Arterial blood pressure control	9	Interruption of treatment
10	Promotion of education and knowledge of the hypertensive patients	10	Modification of life habits
11	Proximity to multiprofessional healthcare teams		
12	Doctor-patient relationship/ communication		

## DISCUSSION

The results of the sample showed that 66.7% of the patients interviewed were females. Most of the women proved to be more compliant with drug treatment and had a lower number of missing doctors' appointments when compared to men. These data are in accordance with the results obtained in a cuban study, in which a greater percentage of patients with full treatment compliance corresponded to the female gender (68.50%)^(8)^.

The individuals studied were between 40 and 80 years of age, more than half of the patients (68.12%) were over 60 years of age, and 24.63% of them were aged between 50 and 59 years. Of those older than 60 years, most stated not recording the time for taking their medications to avoid forgetting. Patients aged between 40 and 59 years and those with ages equal to 80 years were the interviewees that most responded positively to the data collection instrument.

A high age group may have positively influenced the rate of compliance in the population studied, which was also made evident by Busnello et al.^(9)^, who observed that the increase in age was associated with a greater probability of compliance with the treatment recommended. The younger person does not feel as vulnerable to the disease as does the older individual, or may still be asymptomatic, which makes early diagnosis and treatment difficult^(10,11)^.

As to marital status, it is clear that separated individuals have a lower level of compliance compared to those who are single, married or widowed. These last three categories had more than 70% positivity in their responses, showing a greater degree of compliance with the drug treatment when evaluated by the IAAFTR. As a sociodemographic variable, marital status was also analyzed by Karaeren et al.^(12)^, who affirmed that married individuals with AH showed higher levels of treatment compliance (85%) when compared to those who are not married (70%).

Regarding level of schooling, the data showed that the large number of individuals with a low level of education may contribute to the insufficient degree of drug treatment compliance for AH^(13)^. All patients who had completed High School and most of those who had higher education affirmed that they had not missed doctor appointments over the previous 6 months, while only half of the illiterate group could state the same thing.

As to race, both white and non-white individuals achieved similar percentages when interrogated about most of the questions, revealing no difference in the degree of compliance among the races studied.

In analyzing the BMI, recognized as the international standard for evaluating the degree of obesity^(14)^, it was noted that in the population under study, there was a predominance of individuals who were overweight and had abdominal circumference above the standard of normality^(6)^. Similar data were collected in a study performed by Bramlage et al.^(15)^, which identified a strong relation between a high BMI and the abdominal circumference measurement with factors that contribute to blood pressure elevation and development of cardiovascular disease.

Of the patients covered, most presented with controlled SBP and DBP at the time of the interview, and most patients with BP under control responded to the questions positively regarding drug treatment compliance.

Conversely, patients who presented with uncontrolled blood pressure at the interview reached an average of 30.85% of positive responses for the same questions.

When asked about family history of arterial hypertension (father, mother, and siblings), more than half of the patients responded that they had at least one family member with the disease. Literature has shown that a large part of the patients present with some knowledge as to the influence of the hereditary factor in hypertensive disease, a fact that may collaborate to a higher degree of patient compliance with drug treatment, considering knowledge about the disease and family education^(16,17)^.

In classifying duration of the AH and the time of treatment of these patients, it was noted that both those who have had the disease for a short time and those who have been in treatment for a long period had similar answers in terms of drug treatment compliance.

The individuals who affirmed that they earned more than one minimum wage a month had higher indexes of compliance regarding acquisition of antihypertensive medications. The index of individuals with a family income of 3 minimum wages, at the most, had a higher rate of having missed doctors' appointments over the previous 6 months of treatment when compared to individuals with a monthly family income of more than 3 minimum wages.

At the location where the study was carried out, there was an expressive percentage of individuals with a low family income, and many patients mentioned the need for financial help to acquire antihypertensive medications, which may consequently compromise treatment compliance. Thus, the prevalence of hypertension is inversely proportional to level of schooling and income, that is, the higher the level of instruction and economic capacity, the lower the incidence of the disease and the greater the control of blood pressure levels^(18)^.

Students, housekeepers, and those that stated not having any profession/occupation demonstrated higher indexes when questioned about taking medications when they have to leave their homes. This suggests the existence of a strong relation between the frequency of compliance with the drug treatment and the work/ occupation status of the patient, since this last factor may be linked to the presence of obligations, responsibilities, and duties that lead the hypertensive individual to give up care for their disease and the importance of their treatment^(19,20)^.

Those interviewed stated that taking medications at the correct times, the association with daily activities, and the need to carry the medications with them when they need to leave the house or travel are intimately related to family support. However, studies point to the fact that knowledge of a family member about the drug treatment regimen indicated for the patient is unsatisfactory and may hinder the correct compliance with treatment^(21)^.

Most of the patients declared that they provided a new box of medication before the current one runs out. They also reported different forms of acquisition of the medications: sometimes they bought them, sometimes received them for free, and sometimes they got them at the local public healthcare unit that their place of residence is linked to. All the patients affirmed taking the antihypertensive medication correctly, even knowing that their blood pressure was under control.

Most patients denied missing doctors' visits over the previous 6 months of treatment for arterial hypertension. Attendance rate of the patients at the medical appointments may interfere positively in compliance to drug treatment, since the patient's presence with a certain frequency at the unit may offer a reduction in pressure levels, while providing individual motivation, attitudes that contribute towards the reduction of arterial hypertension, a better monitoring of pressure levels, and greater access to information referring to the health/disease process^(8)^.

The survey of factors that interfere in drug treatment compliance included data related to the biosocial and cultural aspects of the individuals, as well as behavioral processes of adaptation and comprehension of the disease and of treatment. A large number of facilitating and complicating factors were found, seeking to establish an analysis of their influence on compliance among hypertensive patients.

The factors of age, gender, and race, according to the understanding of the authors of this study, were considered individual factors, which could be either facilitators or complicators, depending on the characteristic of each person, their life history, their form of facing the disease, and the treatment prescribed.

As to the factors that involve tolerability to the drugs and type of medications prescribed, they were considered facilitators and/or complicators, since both could help or hinder the drug treatment compliance process, as they are related to individual human responses. These factors require patients' knowledge about the medications, dosages, and side effects, especially about perception and understanding of their own organic response.

The greatest use of educational actions referring to motivation and direction towards self-care, besides the establishment of bonds in the patient/healthcare professional relationship as a support basis for implementation of a multidisciplinary and individualized approach to healthcare, may contribute towards the process of drug treatment compliance in individuals with arterial hypertension^(3)^. Additionally, the orientation and choice of drugs with fewer undesirable effects, low cost, monotherapy, dosage convenience, adequate treatment combination, prescription and information in writing for easy understanding, and familiarization of the doctors with therapeutic regimes and treatment for different groups may contribute towards the correct compliance with medications^(3,22,23)^.

## CONCLUSIONS

The data made it clear that, despite the number of facilitating factors for the drug treatment compliance process being greater than the number of complicating factors, more than half the patients interviewed presented with an insufficient level of compliance to use of arterial hypertension medication. This characterizes the need for development of studies focused on the identification of these factors among hypertensive individuals of different ages, genders, races, and social, economic, and cultural levels, in order to favor planning and implementation of health educational systems that allow awareness of healthcare professionals in promoting autonomy, acceptance, knowledge, and adaptation of the patient as to their own disease.
